# Motor Imagery and Motor Execution: A Narrative Review of Electroencephalographic (EEG) Signatures, Methodological Consistency, and Translational Applications

**DOI:** 10.7759/cureus.93011

**Published:** 2025-09-23

**Authors:** Kaviyan Jayalaksshme Srinivasan, Panneerselvam Periasamy, Sasikala Gunasekaran

**Affiliations:** 1 Biological Sciences, Hamilton High School, Sussex, USA; 2 Physiology, Government Erode Medical College and Hospital, Perundurai, IND; 3 Nursing, Government Erode Medical College and Hospital, Perundurai, IND

**Keywords:** brain-computer interface, electroencephalography, event-related desynchronization, motor execution, motor imagery, neurorehabilitation, sensorimotor rhythms

## Abstract

This narrative review evaluates when electroencephalography (EEG) signatures elicited by kinesthetic motor imagery (MI) genuinely approximate those of motor execution (ME), appraises methodological consistency across studies, and outlines pragmatic routes to translation in brain-computer interfaces (BCIs) and neurorehabilitation. A keyword-driven search of Web of Science, Scopus, PubMed, and conference repositories was used to extract empirical, English-language EEG studies reporting sensorimotor rhythm (mu 8-13 Hz; beta 13-30 Hz) event-related desynchronization/synchronization (ERD/ERS) metrics and/or decoding performance for MI and/or ME, with structured extraction of task/sample features, imagery protocol, EEG methods/signatures, MI-ME overlap, translational readouts, and limitations. Across convergent datasets, MI reliably evokes contralateral mu/beta ERD with timing and topography akin to ME, typically with smaller amplitudes and broader fields; realistic decoding benchmarks cluster around the mid-70% for MI versus low-80% for ME, with ≈70% a usability threshold and 15%-30% of naïve users below it. Convergence and performance improve with first-person kinesthetic instructions, higher imagery vividness, synchronised action observation, object-oriented tasks, EMG monitoring, and contingent neurofeedback; source-space modelling and synergy-aware features can lift MI accuracy into the ~82%-95% range in constrained settings, though offline gains often overestimate online control. In stroke cohorts, most patients exhibit clear ERD/ERS, and a meaningful subset exceeds operational thresholds; however, calibration-to-online drops (e.g., ~80% to ~70%) are common and partially recover with adaptive retraining. The principal barriers to translation are heterogeneous protocols (band definitions, referencing, validation), small and selective samples, sparse EMG to exclude covert movement, non-stationarity across sessions, and persistent non-responders. To move from plausibility to practice, future studies should standardise mu/beta windows and baselines, report closed-loop outcomes, personalise training with vividness assessment and synchronised action observation, anticipate drift with adaptive algorithms and periodic recalibration, and integrate MI with robotics, functional electrical stimulation, or virtual reality in multisite trials that track durable functional gains.

## Introduction and background

Motor imagery (MI) refers to the visual or kinaesthetic imagination of a movement without overt execution. Early experimental work showed that imagining a movement engages many of the same sensorimotor networks that underpin actual motor execution (ME), with similar timing and adherence to Fitts’ law [1]. Neural imaging studies reveal that the mental rehearsal of an action activates distributed premotor-parietal circuits and subcortical structures also recruited during ME. These observations support the “functional equivalence” hypothesis: the brain uses shared motor-cognitive processes to simulate actions and prepare for movement. MI is therefore more than a thought experiment; it can induce plastic changes in the motor system and contribute to learning and rehabilitation [2].

Electroencephalography (EEG) offers a unique window into the temporal dynamics of motor cognition because of its high temporal resolution and sensitivity to sensorimotor rhythms. Event-related desynchronisation (ERD), a short-lasting attenuation of ongoing oscillations, is frequently observed in the alpha (8-13 Hz, the mu rhythm) and beta (15-25 Hz) bands during movement generation, observation and imagery [3]. ERD reflects increased cortical excitability and is linked to mirror‑neuron activity, making it an attractive biomarker for detecting MI and ME [4]. Early mobile EEG studies indicate that imagining naturalistic whole-body actions like walking elicits beta and alpha desynchronisation patterns that resemble those recorded during actual walking, particularly in the initial phase of the action. However, MI-induced ERD tends to be spatially broader and of smaller magnitude than ME, suggesting additional cognitive load and variable sensorimotor engagement [1,5].

The translational promise of MI lies in its ability to provide voluntary control signals for brain-computer interfaces (BCIs) and to augment neurorehabilitation without requiring physical movement. Yet real-world deployment faces significant hurdles. Sensorimotor‑rhythm BCIs typically require classification accuracies of ~70% or more to achieve usable control; unfortunately, 15%-30% of naïve users fail to generate distinguishable EEG patterns above this threshold [6]. The disparity between offline accuracy and online performance, non-stationarity of EEG signals, and inter-individual variability (often called BCI illiteracy) limit the generalisability of current findings. Moreover, many MI studies differ widely in sample size, training protocols, frequency bands, and analytical methods, and they often lack direct comparison with ME or proper electromyography monitoring [1,3,4,7,7-11].

Given the above landscape, this narrative synthesis that integrates the biological fit of the EEG signals with study quality is therefore needed to clarify when MI reliably recapitulates ME and how such signals can be translated into real-world systems.

## Review

Database search strategy and extraction

The literature search followed a keyword-driven approach. We constructed a Boolean query encompassing the objective of our review from Web of Science, Scopus and Pubmed: MI and execution (capturing both “motor imagery”/MI and “motor execution”/ME or their synonyms), electroencephalography (EEG) as the measurement modality, sensorimotor rhythms (SMR/mu/beta bands and related event-related desynchronisation/event-related synchronisation (ERD/ERS) phenomena), brain-computer interface or neurorehabilitation applications, and common decoding methods or task descriptors (e.g., classification, power spectral density, common spatial patterns, topographic mapping, kinesthetic or visual imagery). We implemented the search in major scientific databases and conference repositories, without date restrictions, ensuring coverage of both clinical and experimental literature. Titles and abstracts were screened for relevance; we retained only empirical studies published in English that used EEG to measure neural activity during MI and/or execution, reported SMR or ERD/ERS metrics or classification accuracy, and provided sufficient methodological detail to assess quality. Full texts were then examined to confirm inclusion criteria, and data extraction was performed across six domains: (1) sample and task characteristics, (2) imagery protocol (kinesthetic vs. visual, training duration), (3) EEG methods and signatures, (4) MI-ME overlap or divergence, (5) methodological consistency (e.g., sample size, controls, cross-validation), and (6) translational insights (e.g., BCI performance, rehabilitation outcomes). Studies that employed only magnetoencephalography (MEG) or functional near-infrared spectroscopy (fNIRS), lacked EEG‑based measures, or did not meaningfully address MI versus ME were excluded.

EEG signatures: MI versus ME

In the key studies comparing EEG signatures of MI and ME, a consistent pattern emerges: kinesthetic MI reliably evokes mu- and beta-band desynchronisation over contralateral sensorimotor cortex that resembles the response seen during actual movement, albeit often with lower amplitude or broader spatial distribution. Large and statistically rigorous datasets demonstrate this physiological convergence and provide benchmarks for classification performance. For example, a large four-class motor movement dataset showed robust sensorimotor rhythms in both MI and ME, with decoding accuracy reaching 76%-81% and clear evidence that ME still performs better MI in naïve users [12]. High-density EEG studies employing source imaging reveal that kinesthetic MI of hand flexion, extension and rotation recruits premotor and primary motor areas similarly to ME, and that transforming sensor-level data into source space improves classification accuracy by up to 18% [13]. In mobile EEG work on gait, MI of walking produced early beta suppression and rebound patterns closely tracking those of actual walking, with divergences attributable to broader cortical recruitment and higher cognitive load [1]. Event-related designs with high temporal resolution have shown that MI and ME elicit comparable phase-locked and non-phase-locked mu-band responses in primary motor and premotor cortices [14], and larger cohort studies confirm that contralateral ERD/ERS topographies and classification accuracies in mu rhythms are closely matched between imagined and executed finger-tapping, while noting non-trivial inter-individual variability and BCI illiteracy [15,16].

Quantitative analyses of ERD magnitude similarity reveal that vivid kinesthetic imagery predicts stronger MI-ME overlap, emphasising the importance of imagery ability [17], while dynamic causal modelling and connectivity analyses demonstrate that MI and ME share premotor and supplementary motor network engagement, although with distinct directional couplings reflecting greater cognitive control during imagery [18,19]. More recent work using synergy-based decoding of complex grasping tasks reports nearly identical desynchronisation patterns in the 8-30 Hz range for MI and ME and achieves MI classification accuracies exceeding 95%, underscoring the feasibility of translating MI-ME overlap into intuitive brain-machine interfaces [20]. Even fine-grained finger-specific imagery can be discriminated above chance by combining alpha, beta and gamma event-related spectral features, though amplitudes are smaller than for execution [21]. Simultaneous EEG-fMRI (functional magnetic resonance imaging) recordings further corroborate that the magnitude of alpha/beta ERD during MI covaries with blood-oxygen-level-dependent (BOLD) activation in somatotopic sensorimotor cortex in a manner akin to ME, providing a multimodal physiological basis for MI-driven rehabilitation [22].

Finally, data from lower‑limb and posture studies show that MI of sit-to-stand transitions produces clear alpha desynchronisation and movement-related cortical potentials comparable to ME, yielding classification accuracies exceeding 80% and supporting translational applications in gait rehabilitation [23]. Collectively, these studies demonstrate that when imagery is kinesthetic, vivid and well‑practised, MI can recapitulate the spatiotemporal dynamics of ME in EEG, offering robust control signals for clinical BCIs and evidence-based support for MI training in neurorehabilitation. Relevant studies are summarised in Table 1.

**Table 1:** EEG Studies Comparing MI and ME. Abbreviations: ACC, anterior cingulate cortex; ADL, activities of daily living; AO, action observation; AO+MI, action observation combined with MI; AR, autoregressive (model); BCI, brain–computer interface; BOLD, blood-oxygen-level-dependent (fMRI signal); CAR, common average reference; CSP, common spatial pattern; DCM, dynamic causal modeling; DLPFC, dorsolateral prefrontal cortex; ECoG, electrocorticography; EEG-fMRI, simultaneous EEG and functional MRI; EMG, electromyography; ERD, event-related desynchronisation (power ↓); ERS, event-related synchronization (power ↑); ERSP, event-related spectral perturbation; FB-CSP, filter-bank CSP; FgMDM, Fisher-geodesic minimum distance to mean; GLM, general linear model; ICA, independent component analysis; KVIQ-20, Kinesthetic and Visual Imagery Questionnaire (20-item); LDA, linear discriminant analysis; LI, lateralisation index; M1, primary motor cortex; MDM, minimum distance to mean (Riemannian classifier); ME, motor execution; MI, motor imagery; MIQ-RS, Movement Imagery Questionnaire - Revised, Second Version; MMC/SMC, (primary) sensorimotor cortex; MMP, movement-monitoring potential; MRCP, movement-related cortical potential; MVC, maximal voluntary contraction; PCA, principal component analysis; PMC, premotor cortex; PP, (robot-assisted) physical/progressive practice (as described in the source study); PSD, power spectral density; ROI, region of interest; SMA, supplementary motor area; SMR, sensorimotor rhythm(s); SM1, primary sensorimotor cortex; SPD, symmetric positive-definite (covariance matrices); SVM, support vector machine; TFR, time-frequency representation; TPJ, temporoparietal junction; VR, virtual reality; WMNE, weighted minimum-norm estimate.

Authors (Year)	Sample & Task	MI Protocol	EEG Methods and Signatures	MI-ME Overlap/Divergence
Ang and Guan (2015) [24]	125 chronic stroke screened; upper-limb tasks (hand/arm MI; robotic-assisted practice). Three RCTs reported.	Kinesthetic MI of affected limb; structured calibration; MI vs idle; up to 18 sessions (90 min over 6 weeks).	27-ch EEG (NuAmps, 250 Hz), 0.05-40 Hz; features: FB-CSP; bands: mu (8-12), beta (13-30); theta also analysed.	MI evoked reliable ERD/ERS in ~82% of patients; ~60% exceeded >70% accuracy; overlap with ME-like signatures; substantial inter-subject variability.
Sun et al. (2016) [25]	10 subacute stroke inpatients; peg-insertion/removal with affected arm.	Kinesthetic+visual MI; 4 weeks daily. MISAO (MI synchronous with AO) vs MIAAO (MI after AO); 5 s imagery/10 s rest.	C3 (Laplacian); 1000 Hz; 0.05-40 Hz; mu (7-12), beta (17-20); ERD maps/time-frequency.	MISAO produced larger/longer ERD than MIAAO; ERD increases correlated with motor recovery (FMA, PST).
Shuqfa, Belkacem and Lakas (2023) [12]	103 participants (PhysioNet EEG Motor Movement/Imagery); four-class MI & ME (fists/feet).	Kinesthetic MI (limbs); BCI-naïve; multiple runs; no structured MI training.	64-ch (subset 29 SM); preprocessing: 8-30 Hz; Riemannian (MDM, FgMDM); SMR ERD/ERS.	Both MI and ME showed SMR; ME > MI decoding (≈81.5% vs 76.4%); gap attributed to naïve users and weaker MI SMR.
Edelman, Baxter and He (2016) [13]	5 healthy (BCI-experienced); right-hand MI (flex/extend/supinate/pronate).	Kinesthetic MI, self-paced; prior ME priming.	64-ch; Laplacian; 2-30 Hz; features in mu (8-13), beta (13-30); source imaging (WMNE).	Source-space decoding improved MI accuracy (up to +18.6% task-wise; ~+12.7% overall); ERD localised to the contralateral hand area.
Mustile et al. (2024) [1]	21 healthy (final analysis) performing gait; actual 6-step walk vs imagined 6 steps.	Kinesthetic MI (first-person), brief training to focus on leg sensations.	Mobile 32-ch; 500 Hz; analysed 0.1-40 Hz; ERSP; alpha (8-12), beta (13-35).	Strong MI–ME overlap: early beta ↓ and end-beta rebound in both; MI showed broader cortical recruitment (larger distributed beta; stronger early alpha ↓).
Li et al. (2015) [26]	15 healthy; leg flex–extension imagery with/without object; visual observation control.	Kinesthetic MI: object-oriented (OI) vs non-object (NI) vs simple (SI); randomized; no specific pre-training.	128-ch; 2-100 Hz; notch 48-52 Hz; EMG monitored; mu (8–12), beta (18–22); surface Laplacian; Granger connectivity.	OI induced stronger contralateral mu/beta suppression than NI; SI/VO inconsistent; object context deepened motor engagement.
Llanos et al. (2013) [14]	11 healthy men; joystick thumb movements (up/down/left/right): ME, MI, passive.	Kinesthetic MI (feel sensations); no explicit MI training.	128-ch; 512 Hz; Laplacian; mu (8-12); phase-locked (PLr) and non-phase-locked (NPLr) analyses.	MI and ME showed similar mu PLr/NPLr modulations across SM/premotor regions with comparable timing and duration.
Cho et al. (2017) [15]	52 healthy; left vs right hand MI; subset ME trials.	Kinesthetic MI (finger-to-thumb sequences); 5–6 runs with feedback; movement practice included.	64-ch; 512 Hz; EMG recorded; CSP → FLDA; SMR (8-14) ERD/ERS.	Contralateral ERD with ipsilateral ERS in S1; occipital alpha ERD non-discriminative; MI overlapped ME in mu, with visual-processing divergence.
Toriyama, Ushiba and Ushiyama (2018) [17]	20 healthy; isometric wrist dorsiflexion at 10%/40% MVC, then MI.	Kinesthetic MI; vividness via KVIQ-20; first-person; practice and MVC calibration.	5 electrodes over left SM cortex; Laplacian; alpha (7-14), beta (15-35); ERD; ERDsim similarity index.	Higher kinesthetic vividness associated with greater ME–MI ERD similarity (significant correlations); visual vividness not related.
Kim et al. (2018) [18]	20 healthy; sequential finger tapping (ME and MI).	Mixed visual + kinesthetic MI assessed by MIQ-RS; no structured training beyond screening.	32-ch; 0.1-50 Hz; ICA; mu (8–12), beta (13–20); DCM (M1, SMA, PMC, DLPFC).	Overlap: PMC, SMA, DLPFC engaged in both; Divergence: input pathways differed (ME→PMC; MI→SMA) and coupling patterns; MI ERD weaker.
Suwannarat et al. (2018) [27]	11 BCI-naïve healthy; hand open/close, wrist flex/extend, forearm pronate/supinate (MI).	Kinesthetic MI; 8 sessions/task over 4 weeks.	16-ch around C3/Cz/C4; 250 Hz; 8-30 Hz FB-CSP (5 sub-bands); LDA/SVM.	Filter-bank CSP outperformed whole-band; wrist/forearm tasks > hand open/close; most participants improved with training.
Ang and Guan (2017) [28]	6 chronic stroke; MI of impaired hand with robotic haptic knob; repeated feedback.	Kinesthetic MI vs idle; calibration (160 trials) + 18 rehab sessions over 6 weeks.	27-ch; 250 Hz; 0.05-40 Hz; FBCSP in mu/beta; offline adaptive retraining tested.	Calibration 79.8% → online 69.5%; adaptive retraining improved to ~75–77%; ERD/ERS present; session non-stationarity notable.
Pei and Vinjamuri (2025) [20]	10 healthy; six ADL-like hand grasps under ME and MI.	Kinesthetic MI; 2 s prep + 2 s imagery; 30 reps/object; EMG monitored.	45 electrodes (F/T/C/P); 600 Hz; CAR; 3-58 Hz (mu, beta; mu+beta; mu+beta+low-gamma); PCA to 7 features.	Similar 8-30 Hz desynchronization for ME & MI; MI more SMA/pre-SMA localised; decoding high (MI ≈95.5% vs ME ≈89.7%) using beta features (protocol-specific).
Andrade et al. (2017) [29]	20 healthy (EEG) + 1 ECoG patient; imagery of grasping a sphere: self (SMI) vs third-person avatar (TPMI).	Visual MI protocol with VR; self vs third-person contrast.	64-ch EEG (1-100 Hz); ICA; alpha (8-13), low-beta (15-21); ERD/ERS; CSP/PCA; SVM.	Self MI elicited stronger mu/beta ERD; third-person MI showed alpha ERS and temporo-parietal engagement; shared centro-parietal (mirror-system) activity.
Hayashi et al. (2017) [21]	7 healthy males; finger-specific execution vs imagery.	Kinesthetic MI (no overt movement); no extended training.	64-ch; 1024→256 Hz; 0.3-128 Hz; ERSP in alpha (8-15), beta (16-31), gamma (32-128); PCA.	Execution discriminable via beta/gamma and combined features; imagery above chance only with combined (α+β+γ); overlap weaker for imagery.
Zich et al. (2015) [30]	15 healthy; right-hand MI with neurofeedback vs no-feedback during EEG-fMRI.	Kinesthetic MI; real-time feedback blocks.	EEG–fMRI simultaneous; SMR ERD contralateral; artifact-corrected online.	Clear MI-related ERD co-localized with BOLD; stronger modulation with feedback, supporting trainability.
Kaneko et al. (2021) [31]	12 healthy males; AO vs AO+MI vs rest during treadmill walking observation.	AO+MI (imagine self walking in phase); third-person view.	64-ch; 1-200 Hz; ICA; ERSP/PSD (4–40 Hz); alpha (8-12), beta (15-30).	AO+MI > AO in alpha/beta desynchronization over sensorimotor/ACC; phase-dependent modulation resembled actual walking.
Yuan et al. (2010) [22]	13 healthy; ME and MI of hand/foot; simultaneous EEG–fMRI.	Kinesthetic MI (clenching at 3 Hz); EMG monitored; trained.	62-ch; 1-30 Hz; Morlet TFR; alpha (8-12), beta (13-28); minimum-norm sources; 3T fMRI.	Contralateral alpha/beta ERD in both MI & ME co-localised with BOLD; stronger BOLD ↔ stronger ERD (negative covariation); weaker left-hand MI BOLD.
McFarland et al. (2000) [16]	33 adults (incl. SCI/ALS/MS); left/right hand opening-closing: ME vs MI.	MI instruction: “imagine moving” (kinesthetic vs visual not specified); no prior training.	64-ch; Laplacian; AR spectra; mu (8-12), beta (18-25).	Both MI and ME produced mu/beta ERD; MI smaller magnitude; mu ERD contralateral; beta ERD more vertex-diffuse; qualitative MI–ME differences noted.
Yu et al. (2022) [32]	16 healthy; right-hand grasp/lift imagery.	Kinesthetic MI; 20-min imagery with rest cycles; no prior training.	62-ch; 0.5-45 Hz; connectivity via Granger causality; mu (8-13), beta (13-30).	Robust mu/beta ERD during MI; decreased clustering/global efficiency and increased path length/degree; effects fronto-sensorimotor; ERD persisted ~18 s post-task.
Chaisaen et al. (2020) [23]	8 healthy; sit-to-stand and stand-to-sit under AO, MI, ME.	MI implied visual+kinesthetic (video-cued); instruction-based.	11-ch; 1-40 Hz; ICA; alpha ERD during AO; alpha ERS during MI; MRCPs for ME; FB-CSP+SVM.	Stand-to-sit yielded higher classification; distinct MRCPs; MI alpha ERS vs AO alpha ERD underscores condition-specific dynamics.
Eaves, Behmer and Vogt (2016) [33]	14 healthy; rhythmical pantomimed actions; AO+MI synchronized vs static; AO vs MI.	Kinesthetic MI (first-person) per PETTLEP script; synchronized vs static AO+MI.	128-ch; 1-50 Hz; SMR ERD/ERS; ICA/dipole localization.	Synchronized AO+MI produced stronger motor/parietal ERD than AO or MI alone; prefrontal ERD enhanced under synchronized AO+MI.
Pfurtscheller et al. (2006) [34]	9 healthy; MI of right/left hand, both feet, tongue.	Kinesthetic MI; relaxation emphasised.	60-ch; 1-50 Hz; ERD/ERS in 2-Hz bins (6-42 Hz); AAR features; Mahalanobis classifier; κ metric.	Hand MI: robust contralateral mu ERD (~10 Hz); foot/tongue MI: mu ERS (~12 Hz) at Cz/hand area; focal ERD with surround ERS phenomenon.
Van der Lubbe et al. (2021) [19]	14 healthy students; discrete sequence production: executed, imagined, withheld.	Kinesthetic MI (first-person, avoid visual imagery); extended practice.	128-ch; 1000 Hz; Morlet wavelets; theta (3-8), alpha (7-14), beta (12-24); topographic ERD/ERS.	MI showed stronger frontal theta ERS (executive control); ME/withheld showed posterior alpha ERD absent in MI; beta ERD ME>MI>withheld.
Formaggio et al. (2010) [35]	7 healthy; kinesthetic thumb MI inside MRI.	Kinesthetic MI; eyes closed; 1 Hz pacing; EMG monitored.	EEG–fMRI coregistration; 32-ch; alpha (10-12), beta (13-30); artifact-corrected; topographic ERD; BOLD mapping.	Alpha ERD contralateral SM1; beta ERD SMA/central-parietal; BOLD in SMA/SM1/cerebellum; overlap with ME plausible but more diffuse/variable in MI.

Enhancing MI-execution convergence: effects of vividness, action observation, object orientation, and feedback

A higher fidelity between MI and ME appears to be achievable when the MI task is enriched in ways that heighten sensorimotor engagement. Evidence from designs that include both MI and ME shows that kinesthetic vividness plays a central role: Toriyama et al. (2018) [17] observed that participants with higher kinesthetic vividness scores exhibited greater similarity in mu-beta ERD magnitude between imagined and executed wrist movements, suggesting that cultivating a strong first‑person “feel” for the movement narrows the physiological gap between MI and ME. Although many other manipulations have been studied mainly in MI‑only paradigms, their effects are consistent with this principle. For example, synchronous action observation combined with imagery elicits larger and longer-lasting sensorimotor desynchronisation than imagery alone, and these stronger ERD patterns correlate with improved motor recovery in stroke patients [25] and produce phase-dependent modulations that more closely resemble actual gait in healthy participants [31]. Imagining a movement in relation to a concrete object likewise amplifies contralateral mu and beta suppression compared with non-object-oriented imagery [26], implying deeper recruitment of motor circuits. Real-time feedback or neurofeedback can further focus MI-induced rhythms: studies integrating EEG with fMRI have shown that providing feedback during MI increases both ERD amplitude and BOLD activation in motor cortices, indicating that feedback training can steer MI toward execution‑like dynamics [30]. Even the chosen perspective matters - self-referential kinesthetic imagery generates stronger mu/beta desynchronisation than third-person imagery, highlighting the benefit of internal perspective instructions [29]. Taken together, these findings suggest that enhancing MI vividness, coupling imagery with concurrent action observation, incorporating meaningful objects, and providing feedback are promising strategies for increasing MI-ME convergence in EEG measures and improving the translational efficacy of MI-based rehabilitation and BCIs.

Classification and usability benchmarks

Across EEG studies comparing MI and ME, classification performance varies widely, but several patterns emerge. In large datasets with naïve users, MI decoding accuracies hover around the 70%-80% threshold considered necessary for practical BCI control, while ME consistently achieves higher values; for example, a four-class dataset reported median accuracies of 76.4% for MI versus 81.5% for ME [12], whereas MI calibration in stroke patients reached 60% above the same threshold [24]. Targeted studies employing more sophisticated approaches raise these numbers: source‑imaging and synergy-based decoding yielded MI accuracies of 82%-95%, occasionally surpassing ME performance and highlighting the benefit of incorporating anatomical priors and muscle synergies [13,20]. Simpler tasks with minimal training often perform worse-average accuracy in a cohort of 52 healthy adults was 67.5%, with 27% of participants exhibiting non-discriminative signals [15]. Non-standard task design can improve performance: placing a concrete object in the motor task elicits stronger contralateral mu/beta suppression and more reliable classification [26], and individuals with higher kinesthetic vividness show greater similarity between MI and ME‑evoked ERD [17]. Overall, accuracies above 70% are achievable but depend on task complexity, imagery vividness, and advanced algorithms; ME generally remains easier to decode than MI, and inter-individual variability is substantial.

Online stability and adaptation

Evidence for online BCI stability is sparse but revealing. In a longitudinal stroke study, MI classification accuracy fell from 79.8% during calibration to 69.5% during online use, demonstrating that offline benchmarks may overestimate real-world performance [28]. Adaptive retraining partially recovered accuracy (75%-77%), implying that on‑line adaptation and user feedback are essential to counteract session-to-session non-stationarity. Most other reports rely on offline analysis with cross-validation; some note that wrist and forearm MI tasks achieve >70% accuracy with filter‑bank common spatial pattern (CSP), but caution that long sessions induce fatigue and boredom [27]. Real-time neurofeedback paradigms may improve stability: concurrent EEG‑fMRI feedback enhances MI-related desynchronisation relative to no‑feedback blocks, suggesting that continuous sensory reinforcement can help users volitionally modulate mu/beta rhythms [30]. Collectively, these findings indicate that translating MI-based BCIs from offline to online use requires adaptive algorithms, ongoing calibration, and user training; without these, high offline accuracy does not guarantee consistent control.

Translational applications: rehabilitation and assistive BCIs

Large cohort datasets and focused experimental studies together indicate that EEG signatures elicited by kinesthetic MI can be harnessed for functional control and clinical change, while acknowledging clear limits in generalisability. In naïve users, ME remains easier to decode than MI; multi-class benchmarks commonly yield ME accuracies around the low-80% range and MI around the mid-70% range [12], surpassing a commonly cited ~70% operational threshold for basic control but leaving substantial inter-individual variability and a non-trivial subset of non-responders. These observations establish a realistic baseline against which protocol and algorithmic enhancements should be judged.

In clinical rehabilitation, kinesthetic MI reliably produces contralateral mu/beta desynchronisation in a majority of screened stroke patients, and a meaningful subset achieves usable online control. Ang and Guan (2015) [24] reported that ~82% generated detectable ERD/ERS, with ~60% exceeding the >70% accuracy threshold and documented functional gains on the Fugl-Meyer Motor Assessment (≈4.5-7.2 points) when MI-driven control was embedded within structured, robot-assisted practice. Non-stationarity remains a central challenge: performance in the same group dropped from ~79.8% during calibration to ~69.5% online but improved to ~75%-77% with adaptive retraining [28], highlighting the importance of closed-loop adaptation. Timing and sensory context further modulate efficacy. Synchronising imagery with action observation strengthens and prolongs ERD and accelerates recovery in subacute stroke [25], and gait-related protocols show MI beta suppression and rebound that mirror walking, supporting lower-limb rehabilitation [1]; action-observation-plus-imagery paradigms also deepen alpha/beta desynchronisation in phase-consistent ways [31]. Early multimodal pilots coupling imagery with orthoses, functional electrical stimulation, or virtual-reality feedback demonstrate plausible contralateral premotor engagement and suggest training-contingent plasticity, albeit with small samples and modest short-term gains [36].

On the engineering and usability front, specific methodological choices can close part of the MI-ME gap. Transforming scalp signals into source space improves physiological specificity and has yielded double-digit accuracy gains in hand-movement imagery [13]. Decoding strategies that exploit task synergies in the 8-30 Hz range have achieved very high MI accuracies in constrained settings [20], illustrating the value of anatomically and functionally informed feature design. Training across sessions typically improves control for most users [27], and concurrent neurofeedback can amplify MI-related desynchronisation and co-localised BOLD activation [30], offering a route to more reliable volitional modulation of sensorimotor rhythms. These gains, however, are often demonstrated offline, with small, homogeneous cohorts, higher-density montages, and protocol-specific advantages; portability, across-session drift, and cross-subject generalisation remain the main translational bottlenecks.

In practice, successful translation rests on three guardrails. First, protocol quality: prioritise first-person kinesthetic imagery, assess imagery ability (e.g., Kinesthetic and Visual Imagery Questionnaire (KVIQ) and Movement Imagery Questionnaire - Revised, Second Version (MIQ-RS)), pair MI with synchronised action observation when feasible, and provide contingent feedback. Second, methodological consistency: monitor EMG to rule out covert movement; report and standardise mu (8-13 Hz) and beta (13-30 Hz) windows, baseline definitions, spatial referencing (e.g., Laplacian/common average reference (CAR)), validation regimes (subject-dependent vs. cross-subject), and online vs. offline performance. Third, adaptation and personalisation: anticipate non-stationarity with adaptive retraining, titrate task difficulty to user ability, and consider synergy or source-informed features where computational budgets allow. This is how MI-based EEG systems may show credible pathways to clinical rehabilitation and assistive control, provided that decoding claims are anchored in closed-loop performance and that user- and session-level variability are treated as design constraints rather than afterthoughts.

Sports and motor skill training

Outside clinical and assistive contexts, MI-ME EEG paradigms offer insight into motor learning and athlete training. Controlled experiments in healthy adults show that kinesthetic MI can recruit motor and premotor cortices similarly to execution, but with weaker lateralisation and broader cortical involvement, suggesting a plausible neural substrate for skill acquisition. Kraeutner et al. (2014) [37] found overlapping beta‑band desynchronisation in contralateral primary and supplementary motor areas during sequential button‑press imagery and execution, implying that imagery could supplement practice in music or sports training. Eaves, Behmer and Vogt (2016) [33] reported that synchronising imagery with observed actions enhances mu‑beta ERD relative to either modality alone, supporting the use of action observation combined with imagery to reinforce motor representations. However, these studies typically involve small samples, short tasks and homogeneous cohorts (e.g., young university students or elite athletes), and often rely on MEG or low-density portable EEG, making it unclear how findings generalise to diverse populations or translate into tangible performance gains. Moreover, MI-induced oscillatory changes show rapid habituation and are sensitive to imagery vividness and expertise, as seen in studies comparing ballet dancers, other dancers and non-dancers [38]. Thus, while MI appears to engage relevant motor networks during practice, its added value over physical training remains to be established, and the lack of rigorous methodological controls (e.g., randomisation, longitudinal follow-up, blinded assessment) weakens claims of efficacy.

Operational benchmarks for translation (MI vs ME)

Across convergent datasets in this review, real-world expectations are consistent: MI decoding typically clusters in the mid-70% range, while ME sits in the low-80%; a ~70% accuracy operates as a pragmatic usability threshold; and ~15%-30% of naïve users fall below this bar. Advanced pipelines, especially source-space and synergy-aware decoding, can reach ~82%-95%, but these are upper bounds in constrained settings rather than routine performance. Critically, offline calibration often overestimates online control (illustratively ~79.8% to ~69.5%), with adaptive retraining recovering part of the loss into the mid-70s (Table 2).

**Table 2:** Realistic MI-ME Benchmarks for Translation. Treat mid-70% (MI) vs low-80% (ME) as planning baselines; lift MI toward ME via first-person kinesthetic instructions, AO+MI synchrony, object context, vividness training, EMG checks, and neurofeedback. Consider ~70% the minimum viable target for closed-loop control; pre-specify adaptive updates and periodic recalibration to mitigate non-stationarity. Cite ~82–95% as achievable upper bounds in constrained paradigms (source imaging / synergy features), not as expected in broad deployment. Abbreviations: AO, action observation; MI: Motor imagery; ME: motor execution; BCI: brain-computer interface.

Metric	Benchmark	Context / Interpretation
Typical ME accuracy	Low-80%	ME generally decodes above MI in mixed-ability cohorts; e.g., multi-class benchmarks report ME ≈81.5% [12].
Typical MI accuracy	Mid-70%	Achievable in large, naïve datasets; MI ≈76.4% in four-class tasks. Sensitive to task design and vividness [12,15].
Usability threshold	≈70%	Common operational cut-off for basic BCI control; also used in clinical MI-BCI reports [12,24].
Non-responders	~15–30%	Proportion of naïve users who do not reach the usability threshold (“BCI inefficiency”) [6,15].
Offline to online drop	≈10 percentage points	Example in stroke MI-BCI: 79.8% to 69.5% online; highlights session non-stationarity [24].
Upper-bound gains (constrained)	~82–95% (MI)	With source-space or synergy-aware features; treat as best-case, protocol-specific [13,20].

To operationalise the above gaps and ensure that physiologically credible MI-ME signals translate into deployable BCIs and rehabilitation protocols, a concise “Standardisation Starter Pack” (Table 3) is proposed. This table specifies what to standardise, the minimum items to report, and how to anchor claims against realistic MI-ME benchmarks (MI mid-70% vs ME low-80%; ≈70% usability; typical offline then to online drop), so that future studies are both comparable and clinically/engineering-ready.

**Table 3:** “Standardisation Starter Pack” for MI-ME EEG Studies. This table converts review findings into an implementable checklist. “AO+MI” denotes synchronised action observation plus motor imagery, which typically strengthens/prolongs mu/beta ERD relative to MI alone [25,31]. Object-oriented tasks deepen contralateral SMR suppression [26]. Neurofeedback can amplify volitional modulation of SMR and co-localised BOLD [30]. Use mu=8-13 Hz and beta=13-30 Hz as default SMR windows for comparability across studies. Report online (closed-loop) outcomes and the offline then to online drop (e.g., calibration ≈79.8% to online ≈69.5% with partial recovery after adaptation; [28]. Benchmark results against MI mid-70% vs ME low-80% in mixed-ability cohorts and a ≈70% usability threshold [12,15]. Optional upper-bound pipelines include source-space decoding and synergy-aware features [13,20]. Abbreviations: AO, action observation; AO+MI, action observation combined with MI; BCI, brain-computer interface; BOLD: blood-oxygen-level-dependent; CAR, common average reference; CV, cross-validation; EMG, electromyography; ERD, event-related desynchronisation; ME, motor execution; MI, motor imagery; SMR, sensorimotor rhythms.

Domain	What to standardise	Exact items to report (minimum set)
Task and Instruction	Use first-person, kinesthetic MI as default; assess imagery ability; enrich task context	MI perspective; KVIQ/MIQ-RS scores; AO+MI timing (if used); object-oriented task details; feedback/neurofeedback protocol; training dose
Acquisition and Preprocessing	Fixed mu (8–13 Hz) and beta (13–30 Hz) bands; consistent referencing and baselines; EMG mandate	EEG montage; referencing (Laplacian/CAR); filter/band definitions; baseline segment (timing/duration/state); EMG channels, thresholds, and covert-movement exclusion criteria
Outcomes and Validation	Prioritize online (closed-loop) metrics; disclose offline results as secondary; make the gap explicit	Online accuracy/control rate; offline CV metrics; offline then to online delta; validation regime (subject-dependent vs cross-subject); any session-transfer procedures
Adaptation and Personalization	Pre-plan handling of non-stationarity; match difficulty/feedback to user ability	Adaptive retraining schedule; periodic recalibration; recovered gains (if any); difficulty-titration rules; use of source-informed/synergy-aware features (yes/no; brief description)
Benchmarks and Context	Anchor claims to realistic operational thresholds and typical ranges	Statement vs ≈70% usability; placement vs MI mid-70% / ME low-80% norms; cohort composition (naïve vs experienced)
Quality and Transparency	Minimum reproducibility items	Data-exclusion rules; trial counts per condition; artifact handling; preregistered analyses (if any); code/parameters sufficient for replication

Challenges and limitations

Across applications, several recurring limitations temper enthusiasm for translating MI-ME EEG research into real-world systems. Sample sizes are frequently small (often fewer than 20 participants) and demographically narrow, restricting statistical power and generalizability. Many studies lack rigorous controls or randomisation, and only a minority implement EMG monitoring to exclude covert movement. Offline analyses dominate; consequently, reported accuracies may not survive real-time noise, cognitive load or non-stationarities. Classification thresholds vary widely, and a significant fraction of users (10-30 % in some cohorts) cannot achieve sufficient accuracy for functional control, a phenomenon known as BCI illiteracy. Sensor montages range from two to 128 channels, with higher channel counts improving accuracy but reducing practicality. Physiological plausibility is sometimes inferred rather than directly tested; only a few studies collect both MI and ME data to quantify overlap. Finally, clinical studies often lack long-term follow-up; most report short interventions (two to six weeks) and do not assess retention or integration into daily life. Addressing these limitations will require larger, multisite trials, standardised protocols, inclusion of diverse patient populations, closed-loop validation, and integration with complementary modalities such as functional electrical stimulation, robotics and virtual reality.

Future directions and recommendations

To realise the full translational potential of MI-based EEG systems, future research must address both methodological gaps and user-centred design challenges. One priority is to bridge the gulf between offline performance and real-time control by developing adaptive algorithms that can maintain classification accuracy across sessions and users; the adaptive retraining strategy tested in a small stroke cohort by Ang and Guan (2017) [28] is a step in this direction, but larger, multi-centre trials are needed. Harmonising experimental protocols and measurement standards will also improve comparability: consistent use of kinesthetic imagery, EMG monitoring and rigorous cross-validation would help reduce variability across studies. Diverse and patient-oriented samples are essential; most current work is conducted in young, right-handed volunteers, yet BCI illiteracy and inter-subject variability remain significant obstacles. Training programmes that enhance imagery vividness and integrate synchronised action observation, as suggested by Toriyama et al. (2018) [17] and Sun et al. (2016) [25], may boost the proportion of users who achieve usable control. Integrating MI with other modalities such as functional electrical stimulation, robotic exoskeletons, or virtual reality approaches tentatively explored by van Dokkum et al. (2015) [36] could promote neuroplasticity and generalisation to daily living. On the decoding front, synergy-based models and source‑imaging techniques show promise for reducing dimensionality and improving classification accuracy [13,20]. Future systems should also tailor feedback and difficulty to individual ability, perhaps leveraging neurofeedback paradigms like those tested by Zich et al. (2015) [30] to train users to amplify desired neural patterns. Finally, longitudinal studies that track functional outcomes beyond initial training are needed to determine whether gains in ERD/ERS modulation translate into sustained improvements in motor performance, participation and quality of life.

## Conclusions

Taken together, the evidence shows that kinesthetic MI does, in fact, recreate the core electrophysiological footprint of real movement: contralateral mu- and beta-band desynchronisation with broadly similar timing and scalp distribution, though at smaller amplitudes and with wider fields. This resemblance tightens when imagery is first-person and vivid, when it is coupled with action observation, and when users receive contingent feedback. On the engineering side, moving from scalp to source space and using synergy-aware features can lift decoding into the ~82%-95% range in well-controlled settings, whereas large, mixed-ability datasets provide a realistic benchmark (MI in the mid-70% vs ME in the low-80%). In stroke cohorts, most patients express clear ERD/ERS and a meaningful subset crosses the ~70% control threshold, but the common pattern is that offline calibration overestimates online control (for example, ~79.8% dropping to ~69.5%), with adaptive retraining recovering performance into the mid-70s.

What follows is straightforward. MI is a physiologically credible control signal for rehabilitation and assistive BCIs, but it works reliably only when protocol quality, measurement accuracy, and adaptation are built in from the start. The main brakes are familiar: small and selective samples, heterogeneous band definitions and referencing, limited EMG to exclude covert movement, an overreliance on offline metrics, session-to-session non-stationarity, and a persistent minority of non-responders (roughly 15%-30%). Future studies should standardise first-person kinesthetic instructions, assess imagery ability, monitor EMG, predefine mu/beta windows and baselines, and report online-not just offline, outcomes. Practically, systems should expect variability and design around it with adaptive algorithms, user-specific difficulty titration, periodic recalibration, and, where appropriate, integration with robotics, FES, or VR. The question is no longer whether MI can approximate execution in EEG; it is how to make that approximation reliable enough to improve rehabilitation trajectories and day-to-day function.
